# Intravitreal Anti-vascular Endothelial Growth Factor Injection Practices Among Indonesian Vitreoretinal Specialists: Findings From a 2021 National Survey

**DOI:** 10.7759/cureus.116153

**Published:** 2026-09-12

**Authors:** Andrea Silitonga, Hanna Tabita Hasianna Silitonga, Rova Virgana, Erwin Iskandar, Arief Kartasasmita, Grimaldi Ihsan, Made Indra

**Affiliations:** 1 Department of Ophthalmology and Vision Sciences, Universitas Prima Indonesia, Medan, IDN; 2 Department of Public Health and Community Medicine, School of Medicine, Universitas Ciputra, Surabaya, IDN; 3 Department of Ophthalmology, Faculty of Medicine, Universitas Padjadjaran, Bandung, IDN; 4 Department of Vitreoretinal Service, Cicendo National Eye Hospital, Bandung, IDN

**Keywords:** anti-vegf, bevacizumab, intravitreal injections, povidone-iodine, vitreoretinal specialists

## Abstract

Objective: This study aims to describe the preferred methods for performing intravitreal anti-vascular endothelial growth factor (VEGF) injections among vitreoretinal specialists in Indonesia, including pre-injection assessment, bevacizumab preparation, aseptic measures, injection technique, and post-injection care.

Methods: This was a descriptive survey of self-reported practice patterns. A cross-sectional survey was carried out, where active members of the Indonesian Vitreoretinal Society (INAVRS) were asked to complete a 44-item online survey on their intravitreal injection (IVI) technique. The survey was conducted in 2021.

Results: Out of the 116 participating vitreoretinal specialists, the response rate was 82.8%. Bevacizumab was the most commonly used anti-VEGF (73.3%). Around half of the participants (53.4%) assessed systemic and cardiac risks. A large portion (74.1%) did not use prophylactic topical antibiotics. Most injections (92.2%) were performed in a main operating room with the patient lying on their back (96.5%) and using tetracaine eye drops (94%). A majority (78.4%) avoided performing bilateral injections on the same day. Around 60.9% utilized a single puncture method for bevacizumab preparation, and 57.3% stored the opened bevacizumab vial for less than a week (27.5%) to up to a month (24.6%). During the COVID-19 pandemic, 100% of surgeons, assistants, and patients wore masks. Sterile gloves, a lid speculum, and a sterile ophthalmic drape were used by all participants, and 98.3% utilized povidone-iodine for antisepsis. A caliper was used by 69.8% of participants to measure the injection site, and a 30G needle was used in 94.8% of cases, mostly in the superotemporal quadrant (70.7%). Visual acuity post-injection was assessed with a counting finger test by 34.5%, while 95.7% of participants prescribed post-injection antibiotic drops for several days.

Conclusion: Substantial variability was observed in self-reported intravitreal anti-VEGF injection practices, with some divergence from evidence-based recommendations. These findings reflect historical practice patterns and should not be interpreted as evidence of the comparative safety or effectiveness of specific procedural techniques.

## Introduction

The intravitreal injection (IVI) of therapeutic drugs is now one of the most common ophthalmology procedures. Anti-vascular endothelial growth factor (anti-VEGF) injections, such as ranibizumab, aflibercept, and bevacizumab, are now the primary treatment for many retinal diseases, including neovascular age-related macular degeneration, diabetic macular edema, macular edema caused by retinal vein occlusion, and myopic choroidal neovascularization. In 2013, over four million injections were conducted in the United States alone, and it has been anticipated that around six million injections would be provided by 2016 [1].

Several approaches are used in the preparation, procedure, antibiotic administration, and post-injection evaluations for IVIs [1-6]. These procedures often involve steps for preventing endophthalmitis, improving patient comfort, and streamlining clinical workflow. Epidemiological studies estimated that the incidence of endophthalmitis following IVT anti-VEGF injection ranged between 0.029% and 0.058% [7].

Despite the publication of various evidence-based guidelines for IVI practices, it remains unclear how these practices are implemented in the actual world. Researchers in the United States, Europe, Canada, Mexico, India, and Brazil have previously published their recommendations for IVI [8-15]. To date, there have been no reports on these preferences in Indonesia. The purpose of this study is to describe practice patterns, including pre-injection assessment, bevacizumab preparation, aseptic measures, injection technique, and post-injection care among Indonesian vitreoretinal specialists, specifically those who are members of the Indonesian Vitreoretinal Society (INAVRS) and have completed formal vitreoretinal subspecialty fellowship training.

The abstract of this research has already been presented at the 39th Asia-Pacific Academy of Ophthalmology Congress in conjunction with the 49th Indonesian Ophthalmologists Association Annual Meeting in Bali, Indonesia, 22-25 February 2024.

## Materials and methods

This study was a descriptive survey of self-reported practice patterns that was conducted in 2021. Selected active members of the INAVRS who had completed formal vitreoretinal fellowship training (n = 140) were invited to participate in an anonymous survey distributed via email on May 15, 2021.

The questionnaire was based on previously published surveys [7-15] and included additional items relevant to everyday practice. The initial draft consisted of 44 questions covering patient preparation, drug preparation, injection procedures, and post-injection care. The questionnaire underwent expert review by five vitreoretinal specialists from Cicendo Eye Hospital. The experts evaluated the questionnaire for relevance, clarity, comprehensiveness, and clinical applicability. Based on the feedback, the questionnaire was already revised three times to improve wording, remove ambiguity, and clarify the steps of the clinical practice. No formal pilot testing or psychometric reliability assessment was performed. The complete questionnaire is provided as Appendix 1 to facilitate reproducibility.

Respondents were instructed to answer the questionnaire based on their routine clinical practice at the time of the survey, rather than cumulative career experience or isolated cases. The survey was conducted between May 15 and June 15, 2021. Although it was the COVID-19 pandemic, these measures did not substantially alter the standard technique or routine clinical workflow of IVI in participating centers. Accordingly, the reported practices are considered representative of routine IVI practice during that period. 

The survey link was sent to the respondents’ email addresses registered with the INAVRS, which directed them to a web-based questionnaire page on Google Forms (Google Inc., Mountain View, CA, USA), with secure confidential access. The first page of the survey explained the study’s objectives, details, instructions, and the researchers’ contact details. Participants were anonymized, and responses were not visible to other respondents. Each participant was allowed to submit only one response, and questions could be skipped without choosing a response option. Once submitted, there was no opportunity to change the responses. This methodology was similar to the one used in the previous surveys conducted in the USA, the UK, Europe, Canada, Mexico, and India [8-15]. Three reminder emails were sent over a one-month period before closing the survey to non-respondents.

This study adhered to the principles of the Declaration of Helsinki and received approval from the Ethics Committee of Cicendo Eye Hospital with a certificate number LB.02.01/2.3/021/2021 issued on May 5, 2021. All respondents provided informed consent, which was obtained prior to their participation in this study.

Statistical analysis

This descriptive survey study employed univariate analysis, focusing on frequency distributions and percentages, with statistical analysis performed using IBM SPSS Statistics for Windows, Version 25 (Released 2017; IBM Corp., Armonk, New York, United States). As the questionnaire did not require respondents to answer every item, some questions had missing or skipped responses. Therefore, denominators varied across individual questionnaire items. Frequencies and percentages were calculated based on the number of valid responses for each item.

## Results

Demographic features 

The survey was completed by 116 (82.8%) out of 140 retina specialists. The age of respondents ranged from 32 to 76 years, with an average age of 45 years. Of the respondents, 56% identified as male (65/116) and 44% as female (51/116). In terms of experience, 48.3% had been in practice for zero to five years post-fellowship (56/116), 27.6% for six to 10 years (32/116), 17.2% for 11 to 20 years (20/116), and 6.9% for 21 or more years (8/116). Geographically, responses were received from 20 provinces, with the largest number of respondents from DKI Jakarta (19.8%, 23/116), East Java (15.5%, 18/116), West Java (10.3%, 12/116), DI Yogyakarta (7.8%, 9/116), and Central Java (6.9%, 8/116).

Regarding weekly IVI volume, 66.4% (77/116) of respondents performed between one and 10 injections, 26.7% (31/116) performed between 11 and 20, 4.3% (5/116) performed between 21 and 30, and 2.6% (3/116) performed more than 30 injections. Among the anti-VEGF agents used, bevacizumab (Avastin, Roche Diagnostics GmbH, Mannheim, Germany) was the most frequently used by 73.3% (85/116) of respondents, followed by ranibizumab (Patizra, Novartis Pharma Stein AG, Stein, Switzerland) at 19.8% (23/116), and aflibercept (Eylea, Bayer AG, Berlin, Germany) at 6.9% (8/133).

Pre-injection procedures

Table 1 shows the pre-injection procedures that included the prescription of pre-injection antibiotic drops (11.2%, 13/116 of participants), blood glucose and/or systemic blood pressure assessments (53.4%, 62/116 of participants), and the use of topical anesthesia (94%, 109/116 of participants). 

**Table 1:** Pre-injection practices among Indonesian vitreoretinal specialists

No	Questions and Responses	Frequency (n)	Percentage (%)
1	Giving prophylactic antibiotic eyedrops before injection (n = 116)		
	Yes	13	11.2
	No	86	74.1
	Sometimes	17	14.7
2	The class of prophylactic pre-injection antibiotic eyedrops (n = 30)		
	Fluoroquinolone	26	86.7
	Aminoglycosides	1	3.3
	Combination of antibiotic and steroid eyedrops	3	10
3	Systemic evaluation before administering IVI (n = 116)		
	Evaluate blood glucose level/systemic blood pressure	46	39.7
	Evaluate cardiac risk factors	0	0
	Evaluate blood glucose level/systemic blood pressure & cardiac risk factors	62	53.4
	Not evaluating systemic condition	8	6.9
4	Cut-off value of random blood glucose before administer IVI (n = 116)		
	<200 mg/dl	81	69.8
	<300 mg/dl	18	15.5
	Not considering random blood glucose	17	14.7
5	Cut-off value of systemic blood pressure before administer IVI (n = 116)		
	Systolic <140 mmHg, diastolic <90 mmHg	29	25.0
	Systolic <160 mmHg, diastolic <100 mmHg	76	65.5
	Not considering systemic blood pressure	11	9.5
6	Choice of anesthesia for IVI (n = 116)		
	Tetracaine eyedrops	109	94.0
	Lidocaine gel	5	4.3
	Subconjunctival lidocaine injection	2	1.7
7	Timing of administering topical anesthetic medication (n = 116)		
	Less than 5 minutes before IVI	69	59.5
	5-10 minutes before IVI	46	39.7
	More than 15 minutes before IVI	1	0.8

Injection preparation

Table 2 shows the injection preparation. Before obtaining the drug from the vial, 69% (78/113) of respondents cleaned the vial cap with alcohol. Of those preparing aliquots from the bevacizumab vial, 60.9% (67/110) used a single puncture technique. Most respondents (57.3%, 63/110) stored the opened bevacizumab vial, with usage periods varying from less than one week (27.5%, 19/69) to up to one month (24.6%, 17/110).

**Table 2:** Injection preparation practices among Indonesian vitreoretinal specialists

No	Questions and Responses	Frequency (n)	Percentage (%)
1	How do you clean the vial’s stopper/cap? (n = 113)		
	Stopper cleaned with povidone-iodine	19	16.8
	Stopper cleaned with alcohol 70%	78	69.0
	Stopper not given any additional antiseptic	6	5.3
	Others	10	8.8
2	Technique to prepare intravitreal bevacizumab injections for IVI (n = 110)		
	Multiple punctures from the vial	37	33.6
	Single puncture from the vial	67	60.9
	Other: Using an aliquoted one cc syringe (containing 0.1 cc) prepared by hospital pharmacy	6	5.2
3	Do you keep/store bevacizumab vials that have been opened? (n = 110)		
	Yes	63	57.3
	No	47	42.7
4	How long do you keep the bevacizumab vial after opening? (n = 69)		
	Less than 1 week	19	27.5
	1 week	18	26.1
	2 weeks	15	21.7
	1 month	17	24.6

Injection administration

Table 3 shows the administration of IVI. For patients requiring bilateral injections, 78.4% (91/116) preferred not to inject both eyes on the same day. The IVI was performed in the operating room by 92.2% (107/116) of participants. All participants (100%, 116/116) used povidone-iodine for antisepsis before the IVI, with 98% (114/116) applying antisepsis to the conjunctival sac, eyelid, and periocular area. The supertemporal quadrant was the preferred site for injection for 70.7% (82/116) of participants. A 30G needle was used by 94.8% (110/116) of participants. After the injection, 75% (87/116) applied pressure with a cotton swab to avoid vitreous leaking. A majority (62.9%, 73/116) never performed AC paracentesis before or after IVIs.

**Table 3:** Injection administration practices among Indonesian vitreoretinal specialists

No	Questions and Responses	Frequency (n)	Percentage (%)
1	Place of administering IVI (n = 116)		
	Examination/consultation room	0	0.0
	Minor operating room / special room for intravitreal injection	9	7.8
	Main operating room	107	92.2
2	Do you perform bilateral same-day injection if the patient needed IVI in both eyes? (n = 116)		
	Yes, always	7	6.0
	Sometimes, only in certain conditions	18	15.5
	Never	91	78.4
3	If you do inject both eyes on the same day, which of the following applies: (n = 25)		
	I use different vials and medication/supplies on each eye	15	60
	I use the same vials and medication/supplies for both eyes	7	28
	Other	3	12
4	If you do not perform bilateral same-day injection, the timing between the first eye and the fellow eye injection is: (n = 103)		
	1 day	15	14.6
	2-3 days	10	9.7
	1 week	71	68.9
	Other	7	6.8
5	Masks usage before COVID-19 pandemic: (n = 116)		
	Only doctors and nurses using masks	68	58.6
	Doctors, nurses, and patients wear masks	48	41.4
6	Mask usage after COVID-19 pandemic: (n = 116)		
	Only doctors and nurses using masks	0	0.0
	Doctors, nurses and patients wear masks	116	100.0
7	Type of gloves used when doing IVI (n = 116)		
	Sterile surgical gloves	116	100.0
	Non-sterile surgical gloves	0	0.0
8	Do you use eyelid speculum? (n = 116)		
	Yes	116	100.0
	No	0	0
	Sometimes	0	0
9	Do you use ophthalmic drape for administering IVI? (n = 116)		
	Yes, sterile adhesive surgical ophthalmic drape	46	39.7
	Yes, sterile ophthalmic drape linen/cloth	68	58.6
	Not using ophthalmic drape	2	1.7
10	Patient’s position for application of IVI: (n = 115)		
	Sitting position	3	2.6
	Supine position	111	96.5
	Semi-supine position	1	0.9
11	The type of antiseptic used before IVI (n = 116)		
	Povidone-Iodine	116	100.0
	Chlorhexidine	0	0
	Other	0	0
12	What is the povidone-iodine concentration that you use before doing IVI? (n = 114)		
	Povidone-iodine 5% for conjunctiva, 10% for periocular	92	80.7
	Povidone-iodine <5% for conjunctiva, 10% for periocular	15	13.2
	Povidone-iodine 5% for conjunctiva, <10% for periocular	3	2.6
	Povidone-iodine <5% for conjunctiva, <10% for periocular	4	3.5
13	At which sites do you give povidone-iodine before IVI: (n = 116)		
	Periocular skin, eyelid and conjunctiva	114	98.3
	Only on eyelid and conjunctival sac	2	1.7
	Only on conjunctival sac	0	0.0
14	How do you measure the location of injection from the corneal limbus: (n = 116)		
	With caliper	81	69.8
	Not measuring, only approximate	3	2.6
	With marker on the end of trocar	28	24.1
	With caliper and marker on trocar	4	3.4
15	What needle gauge do you use for doing IVI: (n = 116)		
	20 G	1	0.9
	23 G	4	3.4
	30 G	110	94.8
	31 G	1	0.9
16	In which quadrant do you prefer to give IVI? (n = 116)		
	Infero-temporal	7	6.0
	Infero-nasal	3	2.6
	Supero-temporal	82	70.7
	Supero-nasal	11	9.5
	No preference of quadrant	13	11.2
17	Do you perform conjunctival displacement before injecting the needle for IVI? (n = 116)		
	Yes	41	35.3
	No	55	47.4
	Sometimes	20	17.2
18	Your preferred technique to prevent vitreous reflux or incarceration: (n = 116)		
	Place a cotton swab at the injection entry	87	75.0
	Injection with angled scleral tunnel technique	1	0.9
	Place a cotton swab at the injection entry & angled scleral tunnel technique	28	24.1
19	Do you perform anterior chamber paracentesis before or after IVI to prevent an increase in intraocular pressure? (n = 116)		
	Yes, always	4	3.4
	Sometimes	39	33.6
	Never	73	62.9

Post-injection care

Table 4 shows the post-injection care. Most respondents (95.7%, 111/116) prescribed antibiotic drops after the injection, with 67% (77/115) using fluoroquinolones. Visual acuity using a counting fingers test was checked immediately after the injection by 34.5% (40/116) of respondents. In terms of follow-up examinations, 55.2% (64/116) of participants conducted an examination at their office one day after the procedure. 

**Table 4:** Post-injection practices of Indonesian vitreoretinal specialists

No	Questions and Responses	Frequency (n)	Percentage (%)
1	Do you measure visual acuity (e.g., hand movement or counting fingers) immediately after doing IVI? (n = 116)		
	Yes	40	34.5
	No	50	43.1
	Sometimes	26	22.4
2	Do you measure intraocular pressure immediately after doing IVI? (n = 116)		
	Yes	36	31.0
	No	59	50.9
	Sometimes	21	18.1
3	Do you check fundoscopy immediately after IVI? (n = 116)		
	Yes	4	3.4
	No	98	84.5
	Sometimes	14	12.1
4	Do you prescribe prophylactic post-injection antibiotics after IVI? (n = 116)		
	Yes, for several days after IVI	111	95.7
	Antibiotic eye drops are given just one time right after injection	2	1.7
	No, I don’t give antibiotics post-injection	3	2.6
5	The class of post-injection antibiotics that you prescribed: (n = 115)		
	Fluoroquinolone	77	67
	Aminoglycosides	4	3.5
	Combination of antibiotic and steroid eyedrops	32	27.8
	No, I don’t give antibiotics post injection	2	1.7
6	Do you give ocular hypotensive eyedrops/tablets before or after IVI? (n = 116)		
	Yes, always	6	5.2
	Only on certain conditions (ocular hypertension or glaucoma)	86	74.1
	Not giving/prescribe ocular hypotensive medication	24	20.7
7	When do you perform post-injection checkup? (n = 116)		
	1 day after IVI	64	55.2
	2-7 days after IVI	32	27.6
	8-14 days after IVI	5	4.3
	15-30 days after IVI	15	12.9
8	Have you ever had any complication related to intravitreal injection? (Participants can choose more than one complication) (n = 116)		
	Endophthalmitis	24	20.7
	Retinal detachment	5	4.3
	Vitreous hemorrhage	10	8.6
	Retinal vein occlusion (RVO) or retinal artery occlusion (RAO)	2	1.7
	Traumatic cataract (crystalline lens touch)	9	7.8
	Subconjunctival hemorrhage	8	6.9
	Increased intraocular pressure	2	1.7
	Syncope	1	0.9
	Never had any complications	65	56.0

Table 5 shows the comparison of the current survey in Indonesia with recommendations in Europe and practices in the United States of America, Europe, and India.

**Table 5:** Comparison of current survey in Indonesia with recommendations in Europe and practices in the United States of America, Europe, and India AC: anterior chamber; IVI: intravitreal injection; *not available; Ϯpovidone-iodine; ǂanterior chamber; §oculi dextra; •oculi sinistra; Ŧsub-conjunctival; #visual acuity

	Euretina Expert Consensus 2018 [2]	India VRSI Guidelines 2016 [4]	USA 2019 [10]	Europe 2014 [11]	Canada 2014 [12]	India 2020 [14]	This study 2021
Pre-IVI procedures							
Setting							
Physician office	OT, adequate room or in-office setting	OT or procedure room	72	3.5%	72%	N/A*	0
Procedure room			23		N/A*	8%	7.8%
Operating room			5.5	68%	28%	91%	92.2%
Simultaneous bilateral injections	Treat each eye as separate procedure	Not recommended	Always: 71.5%	N/A*	57%	N/A*	Always: 6% Sometimes: 15.5% Never: 78.4%
Pre-IVI antibiotics	Not recommended	A day prior may be beneficial	11%	67%	20%	60%	Always: 11.2% Sometimes: 14.7% Never: 74.1%
Facemask	Recommended	Recommended	33%	N/A*	29%	98%	100%
Gloves	Recommended	Recommended	55%	75%	39%	98%	100%
Lid speculum	Recommended	Recommended	72%	81%	91%	96%	100%
Draping	May not be essential	Recommended	10.9%	82.3%	N/A*	N/A*	98.3%
Anesthesia	Topical anesthesia	Topical anesthesia	Drops: 63% Gel: 32% SC^Ŧ^: 27% Swab: 19% Others: 3%	N/A*	Drops combined with other methods: 90% Drops only: 29% Gel: 25% Swab: 23% SC^Ŧ^: 23%	Local anesthesia:91%	Drops: 94% Gel: 4.3% SC^Ŧ^: 1.7%
Antisepsis	5% PI^Ϯ^ in conjunctival sac	Periocular: 10% PI^Ϯ^. Conjunctival sac: 5% PI	PI^Ϯ^: 97.5% Chlorhexidine: 2%	PI^Ϯ^:90%	PI^Ϯ^: 100%	N/A*	100%
Injection preparation							
Preparing aliquots from bevacizumab vial	N/A*	Single puncture or multiple puncture	N/A*	N/A*	N/A*	Single puncture: 66.2% Multiple puncture: 19.5%	Single puncture: 60.9% Multiple puncture: 33.6%
Time of storage of bevacizumab vial	N/A*	The vial or the aliquots can be stored, kept, and used for a maximum duration of two weeks	N/A*	N/A*	N/A*	A few hours: 39.8% One week: 21.3% Two weeks: 20% One month: 18.9%	Less than one week: 27.5% One week: 26.1% Two weeks: 21.7% One month: 24.6%
Injection technique							
Quadrant	Switch injection sites if patients receive repeated IVI	Any site can be chosen					
Superotemporal			OD^§^ 32% OS^•^30%	N/A*	43%	N/A*	70.7%
Inferotemporal			OD^§^ 62% OS^•^61%	N/A*	63%	N/A*	6%
Superonasal			OD^§^ 1.5% OS^•^4%	N/A *	7%	N/A*	9.5%
Inferonasal			OD^§^ 5% OS^•^5%	N/A*	7%	N/A*	2.6%
Measurement of distance from corneal limbus to site of puncture	N/A*	Sterile calipers	30.5%	N/A*	56%	N/A*	
Needle gauge for intraocular injection	30-gauge or thinner needles are recommended for liquid injections	29/30 G needle					
20G			N/A*	N/A*	N/A*	N/A*	0.9%
23G			N/A*	N/A*	N/A*	N/A*	3.4%
30 G or more			N/A*	N/A*	N/A*	N/A*	94.8%
Method for preventing vitreous reflux	N/A*	N/A*					
Swab compression			N/A*	N/A*	41%	N/A*	75%
Angled scleral tunnel technique			N/A*	N/A*	7%	N/A*	24.1%
Conjunctival displacement	N/A*	N/A*	20.1%	N/A*	11%	N/A*	35.3%
Post-IVI procedures							
Use of antibiotics post-IVI	N/A*	According to the treating doctors	17% instills antibiotics immediately after injection, 9% prescribes antibiotics for home use	89.5%	20%	89% for several days	95.7%
AC^ǂ^paracentesis (pre or post injection)	Might be considered in vulnerable eyes	Routine AC^ǂ^paracentesis is not recommended	14% in patients with advanced glaucoma	N/A*	51%	N/A*	Never: 62.9% Sometimes: 33.6% Always: 3.4%
Verify optic nerve perfusion	N/A*	N/A*	56%	N/A*	48%	N/A*	34% counting finger VA^#^

## Discussion

This study is the first to survey intravitreal bevacizumab injection practices in Indonesia. Comparable surveys have been carried out in countries such as the USA, Europe, Mexico, Brazil, and India [9-11,13-15]. This survey highlights the variability in the practices of IVIs among vitreoretinal specialists in Indonesia.

Only 6% of respondents reported giving bilateral shots on the same day. This was notably different from other nations, such as the United States, where the frequency of same-day bilateral injections ranged between 45% and 71.5% [9,10]. Other nations with greater rates were Canada (57%) and Brazil (36%) [12,15]. Guidelines from the Royal College of Ophthalmologists in the UK, as well as other expert panel recommendations, advise treating the second injection as a distinct procedure from the first. To limit the risk of infection or cross-contamination, use a new set of sterile gloves, fresh povidone-iodine antiseptic, a new sterile caliper, eyelid speculum, and surgical drapes [1-3].

Pre-injection practices

Ranibizumab, bevacizumab, and aflibercept are the most commonly utilized anti-VEGF medicines in ophthalmology because they have a higher affinity for VEGF than natural receptors. Aflibercept binds to both VEGF-B and placental growth factor (PIGF), in addition to VEGF-A isoforms. The incidence of serious systemic adverse effects is rare with all three medications. Specifically, the incidence of arteriothrombotic events and mortality is similar across all three medications. Nevertheless, risks must be considered, particularly in patients with advanced age or cardiovascular and cerebrovascular diseases [1]. When providing anti-VEGF medications to patients with pre-existing cardiovascular or cerebrovascular disorders, the Euretina Expert Consensus advised consulting additional relevant physicians, particularly if these conditions are recent or unstable at the time of the intended IVI [2].

This survey indicated that 53.4% of respondents routinely assessed cardiovascular risk factors before performing IVIs, while 39.7% only checked systemic blood pressure or random blood sugar (RBS) levels. There is little evidence to support the risks of elevated blood pressure or inadequate glycemic control after invasive eye surgeries [16]. The Vitreoretinal Society of India (VRSI) suggested deferring IVIs in cases of uncontrolled diabetes [4]. Seventy percent of the ophthalmologists in this survey considered RBS levels below 200 mg/dl as safe, while 66% required systemic blood pressure to be less than 160/100 mmHg before the IVI. There are no consensus or specific recommendations for systemic blood pressure or RBS cut-off points prior to the IVI. However, glycated hemoglobin (HbA1c) is preferred over RBS as an indicator for ambulatory surgery in the UK [16].

Use of antibiotics

In a 2014 expert panel assessment, it was decided that there was inadequate evidence to support the use of topical antibiotics in IVIs. It is now recognized that topical antibiotic use after the IVI may raise the risk of endophthalmitis or potentially contribute to resistance [1]. The 2018 Euretina Expert Consensus stated, “perioperative antibiotics cannot be considered the standard of care” [2]. Consistent with this evolving understanding, only 11.2% of Indonesian vitreoretinal specialists currently used pre-injection antibiotics. However, a significant proportion of doctors still used post-injection antibiotics (95.7%) for a few days following the IVI (Table 5). The VRSI guidelines stated that “routine use of topical antibiotics for a day prior and three days post-injection may help” [4]. This is consistent with the broad use of topical antibiotics in India (60% pre-IVI and 89.3% post-IVI) [14]. A 2014 survey in Europe reported high usage of topical antibiotics related to IVI (66.7% pre-IVI and 89.5% post-IVI), as well as in Brazil (89.2% post-IVI) [11,15]. Conversely, the trend of using topical antibiotics after IVI showed a decrease in other countries such as the USA (10.9% pre-IVI and 9.1% post-IVI), Asia Pacific (39% pre- and post-IVI), Canada (40% pre-IVI and 33% post-IVI), the UK (30% pre-IVI and 74% post-IVI), and Mexico (22% pre-IVI and 59.4% post-IVI) [10,12,13,17,18]. Quinolones were the preferred antibiotics used by participants in this survey, a preference also noted in retina specialists from Canada and Mexico [12,13].

There is currently enough data to conclude that topical antibiotic use can increase microbial antibiotic resistance and is not associated with a decrease in the incidence of endophthalmitis in the context of IVIs, either pre- or post-injection [1,2]. Several research and professional practice guidelines confirm that taking antibiotics before or after the IVI is unnecessary, and that a thorough antisepsis method is the greatest preventive measure for avoiding endophthalmitis [2,19]. Furthermore, other studies have identified a higher risk of endophthalmitis in antibiotic-treated groups, implying that repeated antibiotic treatment may encourage the establishment of resistant strains, resulting in this paradoxical conclusion [20].

Injection preparation

In this study, multiple doses for different patients were drawn from the same vial when using off-label medications like bevacizumab. Currently, bevacizumab is not offered in single-use vials for ophthalmic applications. On the other hand, approved intraocular drugs like ranibizumab and aflibercept were taken directly from individual vials, with each vial designated for only one patient. Therefore, maintaining aseptic techniques during medication preparation is important. A survey conducted in Mexico reported that 48% of respondents cleaned the vial cap with povidone-iodine and 38.5% with alcohol [13]. In this survey, 69% of participants cleaned the vial cap with alcohol and 16.8% with povidone-iodine. The Indian guidelines that mainly address the use of bevacizumab vials stated that the use of off-label bevacizumab must follow strict aseptic techniques, such as cleaning the rubber stopper of the vial with 70% isopropyl alcohol [4,5].

Bevacizumab is dispensed in larger vials and requires aliquoting for ophthalmic use. This survey found that only 5.4% (6/110) of participants used aliquots prepared by hospital pharmacies. More than half of the respondents (60.9%) used a single puncture technique for aliquoting bevacizumab, while 33.6% used a multiple puncture technique. A previous report noted that 25% of ophthalmologists in the Asia Pacific region perform multiple punctures because their countries lack compounding pharmacies [17]. Das et al. have documented the safety and effectiveness of using single-use bevacizumab vials for up to one week, as long as the vials are refrigerated at four degrees Celsius [21]. There is no agreed-upon standard for how long an opened vial of bevacizumab can be safely used. However, VRSI guidelines recommend that the vial or its aliquots can be stored and utilized for up to two weeks [4].

Protective wear: masks, gloves, and drapes

Various guidelines recommend the use of gloves, masks, and a lid speculum to prevent contamination from eyelashes in the injecting field [1-4]. This survey found that 100% of vitreoretinal specialists in Indonesia followed these instructions. Even more so during the COVID-19 pandemic, this survey found that doctors, nurses, and patients used masks. Similar practices were observed in countries such as India and Mexico, where many doctors used masks and gloves [13,14]. It is interesting to note that the use of masks and gloves was less common in certain countries such as the USA and Canada before the COVID-19 pandemic (Table 5) [10,12]. The decision between sterile and nonsterile gloves is consistent with contemporary office practices, according to a 2014 USA guideline that said that using gloves has not been demonstrated to lower the risk of endophthalmitis [1]. 

It is important to avoid contact between the needle, the injection site, the lashes, and the borders of the eyelids, especially after the last povidone-iodine application. Nevertheless, there isn't enough data to support the regular use of drapes during IVI treatments [1,2]. Therefore, many countries consider that draping may not be essential in IVIs, with only 3-7% of practitioners in the USA and 9% in Canada using drapes [8,9,12]. In contrast, approximately 98.3% of vitreoretinal specialists in Indonesia used sterile ophthalmic drapes. This practice was similar to that observed in Europe (82.3%) and Mexico (82.7%) [11,13].

While operating rooms are not required for IVIs [1,2], more than 92% of ophthalmologists in Indonesia opt to perform them in the main operating room. This is likely due to concerns about the sterility of outpatient clinics and office environments. IVIs are primarily carried out in offices in the USA and Canada [10,12], but in other nations, they are usually carried out in operating rooms or sterile rooms with the same hygienic requirements to reduce the risk of infection, particularly endophthalmitis [13,14,17]. 

According to a meta-analysis, the incidence of endophthalmitis was found to be 0.07% in operating rooms and 0.04% in physicians' offices for IVI procedures [22]. It is noteworthy that a lid speculum and povidone-iodine were used instead of gloves, surgical sheets, or face masks during the procedures carried out in the doctors' offices. However, any location, an operating room, a procedure room, or an office, is suitable for performing IVI. Various elements within different healthcare systems, such as availability, pricing, and logistics, may impact the specific venue chosen [1,2].

Anesthesia

Although there is little evidence to advocate one pre-IVI anesthetic treatment over another, topical anesthesia is widely advised because it is the least invasive strategy [1-4]. The use of ophthalmic drops for IVI anesthesia was the preferred method in Indonesia (94%), Canada (90%), Mexico (97%), and Brazil (67%) [12,13,15]. In other countries such as the USA, 62% of practitioners used anesthetic drops, 32% used topical anesthetic gel, and 26.9% used subconjunctival anesthesia [10]. Subconjunctival anesthesia is a very effective analgesic for IVIs; however, it is typically more painful due to the injection required to give lidocaine in the subconjunctiva. A prior study found that this injection resulted in subconjunctival bleeding in more than half of patients. The use of anesthetic gel is less popular among Indonesian retina experts. However, in the United States and Canada, more than 25% of participants reported using gel either alone or in combination with eye drops [10,12]. The usage of anesthetic gel is debatable, with some publications claiming that administering the gel before povidone-iodine may lower its antiseptic efficiency and allow more germ survival. Other countries' surveys did not mention the period between anesthesia and IVI. In this poll, the majority of respondents gave anesthetic for less than five minutes (59.5%) or between five and ten minutes (39.7%) prior to injection. Proparacaine drops begin to take effect within 20 seconds and last for 10 to 15 minutes, while tetracaine drops begin acting within one minute and last for 15 to 20 minutes [1,2].

Antisepsis

All survey participants reported using povidone-iodine to treat sepsis prior to IVI. This practice is similar to practices in the United States, Canada, Brazil, and Mexico, where over 99% of physicians performed the same antisepsis [9,12,13,15]. Most participants (80%) applied 5% povidone-iodine to the conjunctival sac and 10% to the periocular area. It has been demonstrated that 5% povidone-iodine takes two minutes to eliminate 100% of microorganisms on the surface where it is applied. In addition, this concentration causes less irritation to the ocular surface compared to 10% povidone-iodine [1]. In comparison, 10% povidone-iodine takes four minutes to eliminate 100% of microorganisms. Topical treatment of 5% povidone-iodine for 30 seconds is a safe method of treating sepsis before IVI [2]. Additionally, it is highly recommended that the last substance applied to the ocular surface before injection be povidone-iodine. After the last round of antisepsis, the injection site shouldn't be in contact with the eyelids or lashes [1].

Regarding the application of povidone-iodine, 98% of respondents in this survey used antisepsis on the conjunctival sac, eyelid, and periocular area. The Euretina guidelines state that there is insufficient data to justify preoperative lid scrapes or washes. As a result, it cannot be suggested as a regular and conventional practice [2]. Aiello et al. advised against lid scrubs, suggesting that periocular lid scrubs may increase conjunctival bacterial counts due to displacement from the eyelid margins [20]. But as long as eyelid cleaning or applying pressure to release materials from the meibomian glands is avoided, povidone-iodine can also be applied to the edges of the eyelids and the lashes, as per the opinion of the expert panel in 2014 [1]. A 2014 survey among Canadian retina doctors indicated that 48% used it on the periocular skin and the conjunctival cul-de-sac [12]. However, the VRSI guidelines recommend applying 5% povidone-iodine to the conjunctiva and 10% to the periocular skin [4].

Injection administration

In this survey, 97% of participants reported measuring the exact distance from the corneal limbus to the injection site using a caliper or other measurement device. This is consistent with surveys conducted in Brazil and Mexico (80%) [13,15]. Conversely, more than 60% of retina specialists from the USA did not measure the distance from the corneal limbus to the injection site [10]. Other surveys from Canada and the USA reported that more than 60% of participants preferred the inferotemporal quadrant to perform injection [9,10,12]. In this survey, 70% of respondents preferred the superotemporal quadrant to perform injection. There is no consensus on the precise location or quadrant for IVIs. Most guidelines and expert recommendations allow the injecting physician to decide the quadrant based on the individual patient's needs. For patients requiring multiple injections, it is advised to avoid injecting near previous sclerotomy sites and to alternate injection locations to prevent cumulative vitreous incarceration and the formation of persistent scleral holes [1-4].

The majority of survey participants (94%) used a 30G needle to puncture the eye globe. This data is consistent with polls conducted in the USA, where 61% of respondents likewise used a 30G needle [9]. Liquid injections should be done using 30-gauge or thinner needles, but steroid injections may require bigger needles [2]. In this trial, 75% of participants used a cotton swab to prevent vitreous reflux at the puncture site. Applying pressure with a cotton swab immediately after the puncture helps prevent leakage. Only two surveys, conducted in the US and Canada, reported this technique, with approximately 40% of physicians adopting the practice [9,12].

Post-injection care

In this survey, only 5.2% of participants routinely used ocular hypotensive drops, a rate similar to Mexico (7.5%) and Brazil, where 5% of retina specialists used them. Additionally, 10% of Brazilian physicians prescribed oral acetazolamide [13,15]. Only 31% of participants regularly checked intraocular pressure (IOP) immediately after performing IVI. Current guidelines have stated that routine ocular hypotensive medication is unnecessary. However, it is important to examine IOP before and after IVI [1,2,4-6].

The volume injected during IVI ranges from 0.025 to 0.1 mL. Although comprising only 1-2% of the total vitreous volume, IVI is known to cause both immediate and sustained increases in IOP. Studies show that a healthy eye can typically tolerate short-term postoperative IOP spikes without long-term damage. However, careful IOP control is crucial for patients at risk of vascular optic nerve injury, such as those with glaucoma or a predisposition to anterior ischemic optic neuropathy or retinal vein occlusion. Among diabetic patients, ocular vascular complications like arterial or venous occlusion have been observed in 2-19% of cases following IVI, a rate significantly higher than in the general population [1,23]. High injection volumes and fast injection techniques (more than 0.05 mL in less than one second) may lead to persistent IOP elevation due to injury to the trabecular meshwork [23].

In this survey, just 34% of respondents assessed vision by counting fingers immediately after the injection, a method linked to ensuring proper optic nerve perfusion. This technique is also carried out in the USA (21%-56%), Canada (48%), and Mexico (53%) [9,10,12]. In Canada, specialists reported performing pupil dilation to facilitate a thorough examination post-injection [12]. According to guidelines, the injecting physician must confirm that the patient has functional vision before they leave the clinic or hospital [1,2,5].

Regarding anterior chamber (AC) paracentesis, 5% and 11% of respondents in Canada and Brazil, respectively, performed it in all their cases [12,15]. In Indonesia, 62% of doctors admitted to not routinely performing AC paracentesis. Recent guidelines have shown that AC paracentesis is reserved only for selected cases (such as glaucoma) and not recommended as a routine procedure [1,2,4]. However, in the event of an abrupt IOP rise that causes pain and visual loss, paracentesis in the AC may be performed to release aqueous humor and reduce pressure. Performing a paracentesis may raise the risk of EO [1,2]. Some also recommend considering AC paracentesis if the injected medication is more than 0.05 mL [6].

The risk of negative outcomes from IVI is relatively minimal, which is why there is no consensus on the necessity of post-procedure examinations. In countries such as India and Brazil, 85% and 79% of patients, respectively, received a follow-up examination within the first two weeks after injection [14,15]. In Indonesia, 55% of specialists preferred to see the patient one day after IVI. The VRSI guidelines stated that follow-up may be done three days after injection [4].

This study has several limitations that should be considered when interpreting the findings. First, the potential bias in selecting the participants, as responses were exclusively from active members of the INAVRS. The data were also based on self-reported practices rather than direct observation; therefore, the results are susceptible to recall bias and may not always reflect actual clinical behavior. The findings of this study also represent practice patterns at the time of the survey rather than the current clinical practice; therefore, there may be a rapid evolution of the practice. Similar surveys conducted on a regular basis may reveal changes in preferences as new data becomes available in clinical practice. Finally, these findings should not be interpreted as evidence supporting safety or superiority of specific practices, as the study was not designed to evaluate comparative effectiveness or procedural safety, lacked standardized outcome assessment and procedural denominators, and cannot establish causal or evidence-based conclusions.

## Conclusions

This study indicates variability in IVI administration procedures among Indonesian vitreoretinal specialists. The response rate of 82.8% was significantly higher than in comparable polls done in other nations. The study describes historical self-reported practice patterns and should not be interpreted as validating or endorsing individual procedural practices. The findings cannot determine the comparative safety or effectiveness of different techniques, nor establish causal associations between specific practices and reported complications. Further prospective studies using standardized outcome measures are warranted to evaluate the safety and effectiveness of IVI practices and to assess their alignment with current evidence-based recommendations.

## Appendices

Appendix 1

Intravitreal Injection Practices Questionnaire

Intravitreal Anti-Vascular Endothelial Growth Factor Injection Practices Among Indonesian Vitreoretinal Specialists: Findings from A 2021 National Survey

Instructions: We recognize that individual specialists may perform intravitreal injections across different clinical settings (e.g., private office, academic/public institution, private hospital), which may influence specific techniques. Please answer all questions based on the practice setting that represents the majority (≥51%) of your procedures.

SECTION I. DEMOGRAPHIC INFORMATION

1. Age: _____ years

2. Gender: ☐ Female ☐ Male

3. Current province of practice: __________________________

4. Which vitreoretinal fellowship training program have you completed? ☐ Medical retina ☐ Medical & Surgical retina ☐ I have not completed a fellowship program ☐ Other (please specify): __________________________

5. If you completed a fellowship, how many years have you been practicing in the field of vitreoretina post-fellowship? ☐ 0-5 years ☐ 6-10 years ☐ 11-20 years ☐ >20 years

6. What is the average number of intravitreal injections (IVIs) you perform per week? ☐ 0-10 ☐ 11-20 ☐ 21-30 ☐ >30

7. Which anti-VEGF agent do you most frequently administer for IVI (across all indications)? ☐ Aflibercept ☐ Bevacizumab ☐ Ranibizumab

SECTION II. PRE-INJECTION PRACTICES

8. Do you prescribe prophylactic topical antibiotic eye drops several days prior to IVI? ☐ Yes ☐ No ☐ Sometimes

9. If you prescribe prophylactic topical antibiotics, which class do you select? ☐ Fluoroquinolones ☐ Aminoglycosides ☐ Fixed combination of antibiotic and corticosteroid ☐ Other (please specify): __________________________

10. Which systemic evaluation do you perform prior to the IVI procedure? ☐ Measurement of random blood glucose and/or systemic blood pressure ☐ Cardiac evaluation in patients with identified risk factors ☐ Both of the above ☐ No systemic evaluation is performed

11. What is your cut-off value for random blood glucose prior to IVI? ☐ <200 mg/dL ☐ <300 mg/dL ☐ Random blood glucose levels are not routinely considered

12. What is your cut-off value for systemic blood pressure prior to IVI? ☐ Systolic <140 mmHg, Diastolic <90 mmHg ☐ Systolic <160 mmHg, Diastolic <100 mmHg ☐ Systemic blood pressure is not routinely considered

13. Which type of anesthesia do you utilize for IVI? ☐ Topical tetracaine eye drops ☐ Topical lidocaine gel ☐ Subconjunctival lidocaine injection

14. When do you administer the topical anesthetic agent? ☐ <5 minutes prior to IVI ☐ 5-10 minutes prior to IVI ☐ >15 minutes prior to IVI

SECTION III. INJECTION PREPARATION PRACTICES

15. Prior to withdrawing the medication dose from the vial: ☐ The vial stopper is disinfected with povidone-iodine ☐ The vial stopper is disinfected with 70% isopropyl alcohol ☐ No additional antiseptic is applied to the vial stopper ☐ Other (please specify): __________________________

16. How do you prepare intravitreal bevacizumab injections? ☐ For each patient, medication is directly drawn from the bevacizumab vial (multiple punctures per vial, e.g., 10 punctures for 10 patients) ☐ For a group of patients scheduled on the same day, the total required volume is withdrawn in a single entry (single puncture per vial) and subsequently divided into individual syringes ☐ Other (please specify): __________________________

17. Do you store bevacizumab vials for subsequent use after initial entry? ☐ Yes ☐ No

18. If you answered “Yes” to Question 17, how long do you store the opened bevacizumab vial? ☐ A few hours ☐ 1 week ☐ 2 weeks ☐ 1 month

SECTION IV. INJECTION ADMINISTRATION

19. In which clinical setting do you perform intravitreal injections? ☐ Standard outpatient examination room ☐ Procedure room / Dedicated intravitreal injection room ☐ Operating theater

20. For patients requiring bilateral IVI, do you perform bilateral injections on the same day? ☐ Yes, routinely/always ☐ Occasionally, under selected circumstances ☐ Never

21. If you perform bilateral simultaneous IVI on the same day: ☐ I use separate medication vials and separate surgical instruments for each eye ☐ I use the same medication vial and surgical instruments for both eyes ☐ Other (please specify): __________________________ ☐ I do not perform bilateral injections on the same day

22. Face mask usage policy (PRIOR to the COVID-19 pandemic): ☐ Worn only by the physician and surgical assistant ☐ Worn by the patient, surgical assistant, and physician

23. Face mask usage policy (FOLLOWING the COVID-19 pandemic): ☐ Worn only by the physician and surgical assistant ☐ Worn by the patient, surgical assistant, and physician

24. Type of gloves worn during intravitreal injection: ☐ Sterile gloves ☐ Non-sterile gloves

25. Do you apply an eyelid speculum prior to IVI? ☐ Yes ☐ No ☐ Sometimes

26. Do you place an ophthalmic drape on the patient prior to IVI? ☐ Yes, sterile adhesive surgical ophthalmic drape ☐ Yes, sterile fenestrated cloth drape ☐ No ophthalmic drape is used

27. Patient positioning during IVI administration: ☐ Seated ☐ Supine ☐ Semi-supine

28. Antiseptic agent most frequently utilized: ☐ Povidone-iodine ☐ Chlorhexidine ☐ Other antiseptic

29. If you use povidone-iodine, which concentration protocol do you follow? ☐ 5% Povidone-iodine for conjunctiva, 10% for periocular region ☐ <5% Povidone-iodine for conjunctiva, 10% for periocular region ☐ 5% Povidone-iodine for conjunctiva, <10% for periocular region ☐ <5% Povidone-iodine for conjunctiva, <10% for periocular region

30. Anatomical sites where antiseptic solution is applied: ☐ Periocular skin, eyelids, and conjunctival sac ☐ Eyelid margins and conjunctival sac only ☐ Conjunctival sac only

31. How do you measure the distance of the injection site from the limbus? ☐ Using a surgical caliper ☐ Visual estimation without measuring devices ☐ Using another device (please specify): __________________________

32. Needle gauge used to inject medication into the vitreous cavity: ☐ 20 Gauge ☐ 23 Gauge ☐ 30 Gauge ☐ 31 Gauge

33. In which quadrant do you routinely perform the injection? ☐ Inferotemporal ☐ Inferonasal ☐ Superotemporal ☐ Superonasal ☐ No specific preference

34. Do you perform conjunctival displacement prior to needle insertion? ☐ Yes ☐ No ☐ Sometimes

35. Which method do you employ to prevent vitreous reflux? ☐ Applying pressure to the injection site with a sterile cotton swab ☐ Angled scleral tunnel injection technique ☐ Both of the above ☐ Other (please specify): __________________________

36. Do you perform anterior chamber paracentesis pre- or post-IVI to prevent intraocular pressure elevation? ☐ Yes, routinely/always ☐ Occasionally ☐ Never

SECTION V. POST-INJECTION CARE

37. Do you evaluate visual acuity immediately following the IVI procedure? ☐ Yes ☐ No ☐ Sometimes

38. Do you measure intraocular pressure shortly after IVI? ☐ Yes ☐ No ☐ Sometimes

39. Do you perform funduscopic examination shortly after IVI? ☐ Yes ☐ No ☐ Sometimes

40. Do you prescribe post-injection topical antibiotic eye drops? ☐ Yes, for several days post-injection ☐ Single dose instilled immediately post-injection only ☐ No, topical antibiotics are not prescribed post-injection

41. If you prescribe post-injection topical antibiotics, which class do you select? ☐ Fluoroquinolones ☐ Aminoglycosides ☐ Fixed combination of topical antibiotic and corticosteroid ☐ Topical antibiotics are not used post-injection

42. Do you administer ocular hypotensive agents (topical or systemic) pre- or post-IVI? ☐ Yes, routinely/always ☐ Only under specific indications (e.g., ocular hypertension or glaucoma) ☐ Ocular hypotensive agents are not prescribed

43. What is the timing of the initial follow-up evaluation after IVI? ☐ Day 1 post-procedure ☐ Days 2-7 post-procedure ☐ Days 8-14 post-procedure ☐ Days 15-30 post-procedure

44. Have you ever encountered complications following IVI? (Select all that apply) ☐ Endophthalmitis ☐ Retinal detachment ☐ Vitreous hemorrhage ☐ Retinal vein occlusion (RVO) or retinal artery occlusion (RAO) ☐ Iatrogenic traumatic cataract (crystalline lens touch) ☐ Other complications (please specify): __________________________ ☐ I have not encountered any complications
